# Ethical Implications of eHealth Tools for Delivering STI/HIV Laboratory Results and Partner Notifications

**DOI:** 10.1007/s11904-021-00549-y

**Published:** 2021-03-26

**Authors:** Motlatso Godongwana, Juanita Chewparsad, Limakatso Lebina, Jonathan Golub, Neil Martinson, Brooke A. Jarrett

**Affiliations:** 1 Perinatal HIV Research Unit, Faculty of Health Sciences, University of the Witwatersrand, Johannesburg, South Africa; 2 Programme in Demography and Population Studies, University of the Witwatersrand, Schools of Public Health and Social Sciences, Johannesburg, South Africa; 3 Center for TB Research, Johns Hopkins University, Baltimore, MD USA; 4 Department of Epidemiology, Johns Hopkins University Bloomberg School of Public Health, Baltimore, MD USA

**Keywords:** Contact tracing, Telemedicine, Sexually transmitted disease, Bioethics, HIV, Informed consent, Laboratories, Sexual partners, eHealth, Partner notification

## Abstract

**Purpose of Review:**

eHealth tools are increasingly utilized for communication with patients. Although efficacious and cost-effective, these tools face several barriers that challenge their ethical use in sexual health. We reviewed literature from the past decade to pick illustrative studies of eHealth tools that deliver results of laboratory tests for sexually transmitted infections, including the human immunodeficiency virus, as well as partner notifications. We describe ethical implications for such technologies.

**Recent Findings:**

Our review found that despite widespread research on the use of eHealth tools in delivering laboratory results and partner notifications, these studies rarely measured or reported on the ethical implications. Such implications can be organized according to the four major principles in bioethics: beneficence, patient autonomy, non-maleficence, and justice. The beneficence of eHealth typically measures efficacy in comparison to existing standards of care. Patient autonomy includes the ability to opt in or out of eHealth tools, right-based principles of consent, and sovereignty over healthcare data. To adhere to the principle of non-maleficence, relevant harms must be identified and measured—such as unintentional disclosure of illness, sexual orientation, or sexual activity. Justice must also be considered to accommodate all users equally, irrespective of their literacy level, with easy-to-use platforms that provide clear messages.

**Summary:**

Based on case studies from this review, we developed a list of recommendations for the ethical development and evaluation of eHealth platforms to deliver STI/HIV results to patients and notifications to partners.

## Background

Globally each year, there are an estimated 376 million new sexually transmitted infections (STIs) of *Chlamydia trachomatis*, *Neisseria gonorrhoeae*, *Trichomonas vaginalis*, and *Treponema pallidum* [1]. In 2019, there were 38 million people living with HIV (PLWH) worldwide [2]. Effective control of STIs and HIV depends on widespread and frequent testing of asymptomatic individuals, expedited delivery of testing results, treatment of positive patients, and partner notification and treatment. Though STI/HIV results are typically delivered to patients and their partners via a phone call with a healthcare worker, results are also now being delivered with a variety of mobile- and internet-based technologies [3–5].

Electronic health (eHealth), or the use of information and communication technologies for health, has grown substantially since the late twentieth century, with leaps in the past two decades as personal mobile phones and the internet have become accessible to all income groups worldwide [6–8]. Health services delivered via mobile phones can be categorized into two groups: non-internet mobile services (i.e., phone calls, interactive voice response (IVR), short message service (SMS) texting, and unstructured supplementary service data (USSD)) and internet-based mobile services (i.e., applications (also known as “apps”), messaging apps like WhatsApp, email, social media apps, websites, and online campaigns) [9, 10].

There are multiple benefits of leveraging eHealth to deliver STI/HIV laboratory results to patients and to notify partners of potential exposures. Direct and rapid delivery of results from the lab to patients allows for prompt initiation of treatment and other interventions [11]. Additionally, eHealth tools can reduce patient burden by avoiding clinic visits solely to retrieve results. In resource-constrained settings, this may also reduce clinic wait times and time spent by providers delivering results to patients. eHealth tools can also establish channels of communication—texting, social media, and websites—with traditionally hard-to-reach or historically marginalized populations [3, 7]. These channels have successfully transferred highly confidential data, such as financial information, through services such as M-Pesa and E-Wallet.

There is an abundance of evidence that eHealth tools are effective and feasible for delivering STI/HIV laboratory results to patients, though there are few studies to date on eHealth partner notification platforms [9, 10]. First, text messaging results have shown improved turnaround time from laboratories to providers and from clinics to patients [9, 12]. For instance, in eSwatini, SMSed lab results reached healthcare providers faster and more reliably than paper lab records [13]. Second, eHealth tools increase access to STI/HIV results. For example, in South Africa, a significantly greater proportion (73%) of patients viewed their CD4, viral load, and/or tuberculosis results within seven days via USSD on their mobile phones compared to patients who had to return to the clinic to retrieve their results (8.6%) [14]. Third, patients who receive their results via eHealth also take appropriate follow-up actions, including treatment, in a timely fashion. In an evaluation of the eSexual Health Clinic (eSHC) in England, 92.8% of patients with chlamydia who accessed their STI result collected their treatment, and the median time from notification to treatment was 1 day [15]. A systematic review of eHealth interventions across the HIV cascade showed that delivering test results via SMS to PLWH also reduced time to antiretroviral viral therapy (ART) initiation, especially in settings where transport options were limited [3].

Despite the documented benefits of eHealth tools for delivering sensitive sexual health information, these must be weighed against their harms and other potential ethical considerations, especially as mobile phone usage ownership becomes nearly ubiquitous worldwide. This review was aimed at using illustrative examples from the scientific literature on eHealth tools to describe the current state of technologies, specifically for delivering STI/HIV laboratory results to patients and notifying partners, and then at highlighting areas of ethical concern.

## Methods

We searched PubMed databases to identify and select representative studies that evaluated the present spectrum of eHealth tools and interventions used in the past decade to communicate STI/HIV results to patients and/or their partners. We used variations of the search terms eHealth, health technology, eHealth tools, STI/HIV laboratory results, partner notification, and communication of results to patients. While our search was limited to studies published in English, we included any study with formative, implementation, and endpoint (e.g., efficacy and efficiency) outcomes.

## Results

In total, we chose to highlight fourteen research papers. Papers included results from Nigeria, eSwatini, South Africa, Uganda, Kenya, the United States of America (USA), Peru, England, and Australia. We described the range of interventions (Table 1) and then consider ethical implications using the four major principles of bioethics: beneficence, respect for autonomy, non-maleficence, and justice (Table 2) [25].

**Table 1:** Illustrative examples of eHealth interventions applied for sexually transmitted disease (STI) and HIV laboratory results and partner notification

Intervention name	Technology	Sample (*n*)	Country	Primary outcome measured	Citation	Examples of ethical considerations
STI test result notification						
Healthvana	SMS, email, and web portal	493	USA	Mean number of days between test, notice, and treatment	Cohen et al. [26]	Narrow measurement of benefits
e-Sexual Health Clinic (eSHC)	Web portal	197	England	Access to results and clinical management through the eSHC system	Estcourt et al. [15]	Equal access to data and the internet
Vitira Health platform	Web portal	19	Nigeria	Evaluation of prototype with HIV, hepatitis B, and sickle cell disease test result data	Gbadamosi et al. [17]	Technology not requiring internet access
PRISM	SMS	4081	USA	Proportion of clients that opt to received their gonorrhea, chlamydia, and syphilis results via text message, not clinic visit	Bilello et al. [11]	Risk of unintentional disclosure of results
South Carolina and Mississippi Electronic Acceptability Study	Email and SMS	2719	USA	Acceptability of receipt of STI results via text and/or email	Tripathi et al. [4]	Internet access for email; possibility of spammed emails; risk of unintentional disclosure of results
HIV, CD4, and viral load (VL) result notifications						
LabPush	SMS	1,041	eSwatini	Using SMS to reduce turnaround time for HIV related laboratory results	Jian et al. [13]	Loss/stolen phone threatens confidentiality of lab results sent
MatlaMobile	USSD	400	South Africa	Knowledge of result, appropriate return to clinic, and acceptability	DiAndreth et al. [14]	Phone sharing
Aspect^TM^ HIVST	Mobile app	300	South Africa	Acceptability of mobile app for reporting of HIV and VL results	Gous et al. [18]	Limited language and complicated instructions
CommCare	SMS	138	Uganda	Time from notification to (1) return to clinic and (2) antiretroviral therapy (ART) initiation	Siedner et al. [19]	Confidentiality through messaging variations
NETLAB	Web portal	944	Peru	Awareness of system, enrolment in system, time to publication of test results	García et al. [20]	Technical vulnerabilities with systems to recover lost passwords
HITSystem	SMS	380	Kenya	Proportion of HIV-positive infants who received ART or, if HIV-negative, receipt of all age-specific interventions within 18 months of birth	Finocchario-Kessler et al. [16]	Creative solutions to increase equal access to eHealth platform
Partner notification						
Geosocial Networking (GSN) Apps Acceptability Study	Mobile app	791	USA	Acceptability of mobile app to notify partners of STI results and sexual health services	Contesse et al. [22]	Autonomy of receiving assistance from health departments
inSPOT	Web portal to email electronic postcards (e-cards)	49 500 e-cards across 30,000+ users	USA	Number of e-cards sent, STIs selected, and proportion of e-cards opened by recipients	Levine et al. [23]	Delayed or non-receipt of results
Let Them Know	Email & SMS	2727 SMS, 108 emails	Australia	Evaluation of partner notification website offering email and text message notification services to individuals diagnosed with chlamydia	Bilardi et al. [24]	Autonomy to decide eHealth platform

**Table 2:** Recommendations for the ethical reporting on eHealth platforms to deliver STI/HIV results to patients and to notify their partners

	Definition	Recommendations
Beneficence	Healthcare workers should do all that they can to benefit the patient.	1. Measure and compare effectiveness of the eHealth platform against the standard of care (i.e., the analogue option) delivered in the region. Platforms should be as or more effective than the standard of care 2. Measure and compare the implementation science outcomes (e.g., acceptability, uptake, sustainability) of the eHealth platform against the standard of care delivered in the region* 3. Use unstructured, qualitative data to better understand differences in experiences between patients who receive notifications via eHealth versus the standard of care 4. Stratify results by outcomes (i.e., positive or negative STI/HIV results), as effectiveness may change according to the result received *Because the effectiveness and implementation research outcomes may vary by region, the ethicality of an intervention may also be regionally dependent
Patient autonomy	Patients should have the opportunity to make their own decisions regarding their health.	1. Report whether patients have the opportunity to opt out of eHealth delivery of STI/HIV results and/or partner notification 2. Report whether patient can choose to receive eHealth notifications for negative results but another method for positive results 3. Report whether eHealth platform replaces or supplements in-person opportunities for delivery of STI/HIV results and/or partner notification. If it supplements, describe extent and expediency of opportunities for patient to connect with a healthcare provider through the eHealth platform 4. Report whether the platform requires patient consent before contacting partners 5. Report whether patients have an option to contact partners anonymously 6. Design tools to adhere to local patient privacy laws 7. Though the sale and sharing of non-personally identifying data to third parties is generally not recommended, if this occurs, eHealth platforms should design tools such that patients must opt in (rather than opt out)
Non-maleficence	Healthcare should do no harm.	1. Design eHealth platform to avoid explicitly mentioning sexual health or test results in unsecured messages 2. Report all content received by patients (e.g., text messages) and what security precautions are taken to protect private information 3. Design eHealth platforms to password protect or otherwise restrict access to results, so that they are only delivered to the intended recipient 4. Employ quantitative measures to describe each step of a notification’s lifecycle when delivered via eHealth. This includes but is not limited to (a) the time from when a laboratory enters the result into the platform and when it is received by the patient’s device, (b) the time from when the message arrives in the patient’s device and when it is opened by the patient, (c) proportion of notifications that are not delivered to the device, and (d) proportion of notifications delivered but not read. Compare these metrics to the standard of care 5. Conduct qualitative interviews to discover and describe harms caused by the eHealth platform 6. Measure and report known harms from eHealth platforms: (a) unintentional disclosures of STI/HIV status, (b) unintentional disclosures of sexual activity, and (c) unintentional disclosures of sexual orientation 7. Describe regionally and/or population-specific usage of mobile phones or other platforms and potential for unintentional disclosure (e.g., phone sharing behaviors or prevalence of phone theft) 8. Ensure that multiple forms of contact information are collected as a contingency plan 9. Employ and report on technologies to prevent notifications from being marked as spam 10. Develop features for eHealth partner notification tools to notify patients when a message to their partner(s) remains unopened or unread 11. Limit data storage on electronic databases not managed or regulated by existing healthcare systems
Justice	Healthcare should be fair, with equal distribution of scarce resources and new healthcare interventions.	1. Adhere to standards of human-centered, accessible design to reach persons with low literacy or disabilities 2. Report user-borne cost(s) of the eHealth platform and ranges of incomes among the study population 3. Report regionally specific access to the eHealth platform 4. Conduct formative research of eHealth interfaces and message content with participants who represent a full spectrum of abilities (e.g., native language, literacy, older age) 5. Measure and report comprehension of eHealth message content

### Delivering STI/HIV Laboratory Results

Eleven papers were chosen to describe the range of eHealth platforms that have been used to deliver STI, HIV, CD4, and viral load (VL) results. These included delivery of results via SMS, USSD, mobile applications, and web portals [4, 11, 13, 15, 16, 18–21, 25, 26]. Some eHealth platforms delivered binary results (negative or positive) for STI/HIV tests directly to patients. For example, the USA-based Healthvana notified patients via SMS or email that their STI results were ready; patients then logged into a web portal to look at their result [26]. Platforms varied in whether they required a password to access results. Other platforms notified patients that results were ready but required an in-person visit to retrieve them. For example, the HITSystem in Kenya sent an SMS to notify mothers when their infants’ HIV results were ready but did not provide the result itself in the message [16]. Some systems provided interpretation of the results to assist patients in taking the appropriate next action. For example, CommCare in Uganda delivered CD4 test results to patients and indicated when the count was abnormal to prompt return to the clinic [19]. Most platforms supplied information to multiple users. For example, NETLAB in Peru communicated HIV results to laboratory personnel, health providers, and PLWH [20]. Lastly, some eHealth platforms included educational information so that patients could learn more about their laboratory results, independent of their healthcare provider [11].

### Notifying At-risk Partners

After a patient is diagnosed as being positive for an STI, they may proactively disclose their recent partners to their healthcare provider or their provider may prompt the patient to list their partners. Partner notification is the process of informing the patient’s sexual partners that they may have been exposed to an STI, educating the partner, supporting the partner in seeking testing, and if necessary, providing treatment. Traditional methods of partner notification include patient referral, where patients inform partners themselves; provider referral, where healthcare workers notify the partner without disclosing the original patient’s identity; and contract referral, where patients agree to notify partners and healthcare workers get involved if notifications are not completed by an agreed time frame [21].

Three papers were chosen to describe the range of eHealth platforms that have been used to notify partners of STI/HIV results [23, 24, 27]. Technologies included mobile apps, SMS, email, and electronic postcards (e-cards), which are digital greeting cards that can be personalized for specific recipients to receive via email. eHealth platforms for partner notification worked in a variety of ways. In the Australian app, *Let Them Know*, patients used an SMS or email notification to message partners about their STI status directly or anonymously [24]. A similar platform in the USA, inSPOT, allows people to select an e-card from six pre-specific designs, add an STI, write an optional message, and—either naming themselves or anonymously—send it to partners they may have exposed [27]. Recipients receive an email with information about the specific STI and testing sites, with more than 49,500 in the years 2004–2008. In a formative study in the USA, men who have sex with men (MSM) reported a preference for notifying their partners on their own but did not mind being notified by the health department through geosocial networking apps [23].

## eHealth Tool Ethical Considerations

### Beneficence

Beneficence is the principle that healthcare workers should do all that they can to benefit the patient. eHealth interventions should only be used if the evidence demonstrates that these technologies are equal or better than the equivalent face-to-face encounter. The primary purpose of every eHealth tool presented was to describe the hypothetical or actual benefit to patients; however, not all papers rigorously weighed these benefits directly against the benefits offered by the standard of care. For example, the Healthvana intervention performed a relatively unbiased pre-post study to show that time to notification and time to treatment decreased; however, there was no discussion of what in-person care benefits may be lost [16]. There are few studies to date on the efficacy, effectiveness, and efficiency of eHealth platforms for partner notification, which make it difficult to consider potential benefits and harms to determine its ethical balance. STI/HIV results communicated through SMS or phone call are often short and discreet, making it difficult for providers to gauge the receiver’s response [27]. For the patient, lack of in-person contact may cede the opportunity to ask and receive additional information or agree to a follow-up visit [28]. While sending only negative results which do not require or prompt for a response may be of benefit to some patients, others may prefer to receive positive results within the comfort of their homes and surrounded by people that can support [29]. For example, compared to the standard of care, users of the MatlaMobile in South Africa preferred to receive their CD4, viral load, and/or TB laboratory results via USSD [14]. Given this, it is important that the communication of results using eHealth tools be tailored for each patient in order to maximize the resulting benefit or the patient.

### Autonomy

Autonomy is the principle that patients should have the opportunity to make their own decisions regarding their health. Therefore, eHealth should not replace face-to-face contact unless the patient actively opts in. Patients also need to be able to opt out of receiving their results via eHealth at any time and to choose to receive different results via different channels. For example, the acceptability of eHealth tools to deliver negative and positive results may vary by demographics and result. In the 2012 South Carolina and Mississippi Electronic Acceptability Study, more than 60% of 2500 clinic attendees in the USA indicated that they would prefer to receive their STI results by SMS or email, regardless of whether it was positive or negative [4]. Electronic delivery was more popular among young people. In the Vitira and PRISM studies, participants preferred to only receive negative STI/HIV results through SMS and/or phone call but preferred an in-person visit to receive positive results [11, 18]. Participants should also be able to choose how to receive their result. For example, in the CommCare study in Uganda, participants were randomized to receive one of several messages, including coded messages (e.g., “ABCDEF” or “The big game is here — your tickets are ready!”) that were explained to the patient during enrolment and blunt messages (e.g., “This is an important message from your doctor. You had an abnormal test result.”) [19]. Given all messages tested were effective, patients should be given choice to, for example, protect their privacy.

Partner notification systems are viewed cautiously by vulnerable populations due to violations of privacy, breaches in confidentiality, and coercive medical practices [30]. The WHO attempts to address such issues by publishing guidelines on right-based principles of consent, confidentiality, counseling, correct test results, and linkage to care for all HIV testing and partner notification (WHO, 2016). However, the principle of autonomy should allow for patients to first opt into sharing results themselves with their partners and then, per contract referral guidelines, consenting to have healthcare workers get involved if notifications are not completed by an agreed time frame. Patients should also have the opportunity to choose the platform that they use to notify their partners and to receive help from health departments. In a focus group from the GSN study, for example, MSM were comfortable receiving and sending messages about potential STI/HIV exposures on location-based dating apps (e.g., Grindr) and were interested in opting into being able to do so anonymously with help from health departments [23]. However, the autonomy to send messages anonymously must be weighed against the possibility of misuse or abuse of eHealth platforms. For example, the Let Them Know platform allowed anyone to choose sending either an SMS or email to alert recipients of a potential STI/HIV exposure [24]. However, as noted by the authors of the Let Them Know study, there was no way to confirm that senders had truly been diagnosed [24]. Allowing patient autonomy in how they notify partners may also need to be weighed against the effectiveness of each modality. For example, the inSPOT study reported that 20–40% of partner notification e-cards were ever opened by recipients [27].

In some settings and populations, HIV testing and partner notification are mandatory or enforced, such as for gay men, migrants, people who use drugs, sex workers, and transgender people [30]. Some governments are given augmented authority or presume healthcare workers to have this authority to identify and contact partners without patient consent [30]. Socially marginalized and/or criminalized groups commonly have their ability to provide informed consent rejected as well as their privacy and confidentiality violated, therefore undermining the principle of individual autonomy. The consequences of the patient’s rights being violated include criminalization and increased risk of violence by neighbors, co-workers, and healthcare providers. HIV testing and partner notification without consent or assurances of confidentiality are unethical and undermine healthcare-seeking behavior. Stigma associated with diagnosed HIV and public disclosure about one’s sexual and/or drug using practices result in loss of jobs, health insurance, homes, social connection, and support services [30]. Proper patient information and education are important to ensure patients understand the risk of eHealth. It is essential for populations that are marginalized by such information to be engaged in the design, implementation, and evaluation of partner notification services [30].

### Non-maleficence

Non-maleficence is the principle that healthcare should do no harm. Non-maleficence should be considered a guiding principle when eHealth is used to notify patients of their STI/HIV results and partners of their contact with someone who has tested positive.

#### Delayed or Non-receipt

In face-to-face contact, providers are better assured that patients received their STI/HIV results after testing with an immediate guide to next steps. However, a major limitation of eHealth tools for communicating STI/HIV laboratory results is the risk of results not reaching the patient. SMS or emails with positive STI/HIV results may be sent but are delayed or not received because of limited or no network coverage, spam filters, or incorrect contact information. For example, the HITSystem in Kenya found that 27% (*n* = 399/1495) of text messages were never received by mothers living with HIV who were waiting to be notified about their newborns’ HIV tests and wellness [21]. In another example, the inSPOT study found that e-cards for partner notification required regular changes to their subject lines to keep messages out of spam filters [23]. Despite this, only a minority of e-cards were ever opened. Both delays and non-receipt of STI/HIV results place patients at risk of missed diagnosis and initiation to treatment, which may contribute to advanced morbidity [31]. Patients with delayed STI/HIV results can also continue transmission without their knowledge. Additionally, if test results are not received via the eHealth platform, patients may not have the benefit of immediate STI/HIV counseling which could increase anxiety. To mitigate the risk of delays or non-receipt, healthcare providers using eHealth tools to communicate with patients may wish to collect multiple ways to contact patients and employ spam-avoidance technologies. eHealth tools for partner notification should consider implementing a method to let patients know that their message to their partner(s) has remained unopened or unread.

#### Unintentional Disclosure of STI/HIV Status, Sexual Orientation, or Sexual Activity

The use of eHealth tools for communicating STI/HIV results poses challenges to maintaining confidentiality. STI/HIV test results should be kept confidential [32]. A breach in ethics surrounding confidentiality may arise where either negative or positive STI/HIV results are received by another person. For example, in the HITSystem study, several mothers did not have mobile phones and opted to receive updates on the phone of a local healthcare worker instead [21]. However, patients may also share a phone with their spouse or others within the household. This may be problematic if a person other than the patient is in possession of the phone when an SMS with a positive STI/HIV result is received. For this reason, some eHealth tools included initial, non-password-protected messages written in neutral language that did not explicitly mention STI/HIVs or other health-related information. For example, the notification message from the MatlaMobile study message was “Please dial 1111 to view your results from MatlaMobile” [14]. Others alluded to healthcare, “Please bring baby to the clinic” [21] and only delivered results once in-person. Of the eleven STI/HIV eHealth tools included in this review, five required patients to enter a password before accessing the results [13–15, 17]. Only the NETLAB system in Peru reported a system for recovering usernames and passwords [20]. If the recovery of usernames and passwords is unregulated by healthcare workers, however, this could lead to unauthorized access to health results by persons close to the patient, who may know enough to be able to pose as the patient. Unintentional disclosures were measured in a handful of studies, including the MatlaMobile and HITSystem studies, in which there were no unintentional disclosures reported [14, 21]. Yet, unintentional disclosures do occur, as in one study assessing the acceptability of an SMS program to improve ART adherence, in which 3% (*n* = 3) of participants reported unintentional disclosures of their HIV status [33]. Unintentional disclosures may be more common when people share phones or when phone theft is common. For example, in South Africa, at least 12.4% of participants reported sharing a phone with ≥1 other person and nearly 60% reported needing to replace their phone in the past year [34].

Partner notification services have been described as being difficult and uncomfortable, inducing fear of unexpected reactions and potential repercussions to the relationships especially after HIV status disclosure [35]. Partner notification services are further complicated when having multiple, concurrent sexual partners, which may lead to unintentional disclosure about sexual activities [30, 35]. Reports of harm resulting from partner notification were not present in the studies included in this review. However, this does not preclude the stigma, discrimination, violence, blackmail, and other negative connotations that may be associated with receiving a result [30, 36]. Lack of clearly articulated mechanisms for reporting negative experiences and lack of legal provisions to protect people diagnosed with STIs/HIV against potential harm exacerbate the issue [30].

#### Data Security and Storage

Lastly, data security may be at risk if patient information is entered, sent, or stored outside of a protected electronic health record system. For example, in eSwatini, the LabPush platform allowed laboratories to send clinic nurses STI/HIV results but relied on the nurses to delete the notifications after capturing it onto their health records. In cases where the nurses forgot to delete results or the phone was lost, patient health information remained accessible to anyone in possession of the phone [13]. To address this issue, MatlaMobile leveraged a USSD system, which is what most e-banking is conducted on [15]. USSD allows information to be accessed and viewed but not stored on a recipient’s phones. For partner notification systems, both the inSPOT and Let Them Know servers intentionally did not store any contact information entered after messages had been sent [24, 27]. Newer eHealth innovations ought to address concerns of confidentiality and security when health information such as test results is sent from laboratories/providers to patients.

### Justice

Justice is the principle that healthcare should be fair, with equal distribution of scarce resources and new healthcare interventions.

#### Equal Access for Low-Literacy Users

eHealth platforms should be as interactive as possible, lending themselves to be personalized and adaptable as circumstances of intended users change [37]. For example, laboratory results communicated through eHealth platforms need to be accessible and presented in simple and clear language in order to also accommodate recipients or end users with low literacy levels [38]. In the evaluation of the Aspect^TM^ HIVST app, some participants (1.3%, *n* = 4) requested additional languages to be used while others (1.7%, *n* = 5) suggested that instructions be made simpler to understand [18].

#### Equal Access for People Without Phones or Internet Access

Accessibility in this regard extends to overcoming the digital divide and ensuring that all intended recipients have equal access to internet/data connectivity and are proficient in the health technologies being used. Costs of data must be considered, as these may be expensive and unaffordable for low-income earning patients thus delaying or hindering successful delivery of results. For example, of the 197 eligible participants included in the eSHC system, 161 accessed their results online [15]. eHealth tools can be used where health providers are confident that patients can have access to a phone, tablet, or laptop in order to access results. While this may be difficult to achieve in the context of existing socio-economic inequities, particularly in low-resource settings and where there are limitations to accessibility, alternate platforms of communicating results could be used. For example, the Vitira Health platform in Nigeria was developed to allow both patients and providers access to HIV, hepatitis B, and sickle cell disease test results without internet connectivity [17]. The HITSystem in Kenya allowed patients to appoint a healthcare worker’s phone to receive results on their behalf [21]. An important way to overcome some of the barriers to establishing interactive digital health systems is through the use of user-centered design [39]. This includes taking into account the user’s preferred language of communication and the method of preference to receive results or notifications (e.g., face-to-face, SMS, phone calls, email, or a combination or methods). Employing these methods and aiming to understand the user’s requirements in the communication of health-related results and notifications have shown to lead to improved levels of interaction and user acceptability [39].

## The Future of eHealth Tools in Delivering Laboratory Results and Partner Notifications

There are several technologies on the horizon that will substantially change the landscape of eHealth for delivering STI/HIV laboratory results and partner notifications. These advances will not only increase the advantages, and hence lure, of the technologies but will also reshape relevant ethical considerations.

For example, chatbots—or automated conversational agents—are increasingly being used to allow patients to message back and forth with a computer that uses automated scripts or natural language processing to respond. To date, this interactive technology has been used to engage patients on topics such as HIV testing, prevention, and management [40–42]. Chatbots commonly leverage SMS or messaging apps such as WhatsApp, which allow for end-to-end encrypted messaging. They are able to provide useful information when healthcare providers are unavailable (e.g., after clinic hours) or the patient prefers not to discuss with a healthcare provider (e.g., too anxious or embarrassed). However, a recent study showed that less than half of the surveyed population, 257 sexual and reproductive clinic users in UK, found chatbots favorable possibly due to concerns about perceived lack of confidentiality, empathy, and security [43]. Regulation of chatbots and mobile applications is therefore essential. As chatbots become more common, their ethical implications for delivering STI/HIV laboratory results and to notify patients will need to be considered.

Another emerging technology is biometric signatures—such as fingerprint, voice recognition, or iris scanning. These technologies offer the benefit of verifying the identity of a user before granting access to results. For example, a study in South Africa showed that participants perceived these technologies to be useful in overcoming security concerns, such as only allowing an intended recipient to receive or access ART [44]. However, questions arise about how such highly identifiable data would be stored, protected, and used. These considerations are currently outside the scope of this paper but should be explored later.

## Discussion

This review found evidence that eHealth is an efficacious method for delivering STI/HIV laboratory results the point of care to patients, for partner notification, and to provide sexual health information. However, ethical challenges persist and were not well measured in most eHealth studies.

Health systems adopting eHealth interventions often applied a blanket approach, assuming that all intended users will have internet access, no connectivity issues, and prefer electronic communication over the traditional face-to-face contact for result notification [45–47]. Most studies where acceptability of eHealth for result notification was shown to be high were conducted among users in urban communities, often undermining challenges pertaining to potentially reduced phone or internet access in rural areas [48]. In this review, only one study demonstrated that users did not need internet connectivity to access STI/HIV results on a mobile application [17]. While this particular application was designed for clinicians, it holds promise to overcome some of the connectivity challenges experienced in geographically isolated areas or among clients who cannot afford the connectivity costs.

Even in geographical areas where eHealth is preferred and easily accessible, notable limitations to delivering positive STI/HIV test results through eHealth include results sent at an inconvenient time (e.g., while recipient is at work) and in the absence of someone to provide emotional support [49]. Where SMS has been used to communicate results, one study demonstrated that participants who did not receive an SMS often misinterpreted that this meant their results were positive [19]. In this review, we raised several other issues including concerns over phone sharing, fear of losing a phone with confidential information, the lack of empathy in relying on technology to send results, and delayed or non-receipt of results. For partner notification, communicating results without the consent of patients and partners ignoring results are also of major concern. eHealth tools should be mindful of individual circumstances and preferences.

The future of eHealth tools in delivering STI/HIV laboratory results and partner notifications relies on their ability to be adaptable. The reduced confidentiality of eHealth tools remains a concern. For example, the information sent through SMS and email is generally unencrypted and can theoretically be intercepted by anyone aside from the client or the client’s partner [50]. To overcome some of the privacy concerns, new technologies should consider incorporating security features such as biometric signatures to ensure that the recipient’s identify is verified prior to sharing results. Additionally, consent to have results communicated through eHealth should be obtained from patients. Results sent through eHealth tools should be short, concise, and in the language of the user for ease of interpretation. However, it is important for senders to prompt the recipient to take action (i.e., return to clinic), especially when positive results are communicated. Laboratories or clinicians relying on eHealth tools to communicate positive results can further create a time frame for patients to respond and have measures in place for follow-up. In general, eHealth should aim to supplement and not replace traditional methods of accessing results or partner notification, as per many patients’ preference [51].

## Conclusion

Ethical implications are important to consider prior to the design and implementation of any eHealth system. We suggest that eHealth systems consider the principles of beneficence, patient autonomy, non-maleficence, and justice to mitigate some of the ethical concerns presented here, such as the delay or non-receipt of results, results sent to an incorrect person, or results communicated to a patient’s partner without their consent. Given the growing acceptability of eHealth for STI/HIV laboratory results and partner notification, we recommend that eHealth platforms improve their security features to overcome concerns over the lack of privacy and confidentiality in sharing results using eHealth.
